# Emergency and Non-Referral Admissions as Predictors of Hospital Mortality Among Adults with Congenital Heart Diseases: A Nationwide Claim-Based Registry Study in Japan

**DOI:** 10.3390/healthcare14030315

**Published:** 2026-01-27

**Authors:** Yoshihide Mitani, Michikazu Nakai, Isao Shiraishi, Hiroyuki Ohashi, Hirofumi Sawada, Hideo Ohuchi

**Affiliations:** 1 Department of Pediatrics, Mie University Graduate School of Medicine, 2-174, Edobashi, Tsu 514-8507, Japan; 2 Department of Statistics and Data Management, University of Miyazaki, Miyazaki 889-1692, Japan; 3 Department of Pediatric Cardiology, National Cerebral and Cardiovascular Center, Suita 564-8565, Japan

**Keywords:** adult congenital heart disease, healthcare transition, epidemiology, healthcare policy, universal healthcare coverage, claim data

## Abstract

**Background:** Improved pediatric cardiac care has markedly increased the adult congenital heart disease (ACHD) population worldwide, creating new clinical and healthcare delivery challenges. However, nationwide evidence on predictors of acute outcomes in ACHD patients, particularly the impact of disrupted specialist care under universal healthcare systems, remains limited. **Methods:** We conducted a retrospective analysis using Japan’s nationwide administrative database from 2013 to 2022, evaluating hospital admissions of ACHD patients aged ≥15 years. Patients were categorized into surgical, catheter-based, and medical treatment groups. Multilevel logistic regression models identified predictors of in-hospital mortality, including emergency and non-referral admissions as indicators of impaired continuity of specialist care. **Results:** A total of 27,754 admissions were analyzed (median age 59 years; 49% male). Emergency admissions accounted for 35.2%, non-referral admissions for 9.9%, and overall in-hospital mortality was 5.0%. Older age, admission to non-ACHD centers, higher CHD complexity, emergency admissions, and non-referral admissions were independently associated with increased mortality. In addition, older age, CHD complexity, and admission to non-ACHD centers predicted emergency and non-referral admissions. **Conclusions:** These findings show persistent gaps in specialist care continuity for ACHD patients despite universal healthcare coverage and support the need for integrated ACHD care networks to improve outcomes in this aging population in Japan.

## 1. Introduction

Advances in pediatric cardiology and congenital heart surgery have markedly increased survival among children born with congenital heart disease (CHD), resulting in rapid growth of the adult congenital heart disease (ACHD) population worldwide [1,2,3,4,5,6,7,8]. Currently, adults constitute approximately two-thirds of the entire CHD patient population in developed countries, and their numbers are expected to continue rising [9,10]. As this population grows and ages, new clinical challenges emerge, particularly managing complex cardiovascular comorbidities such as heart failure, arrhythmias, coronary artery disease, and endocarditis, which require lifelong specialized care [11,12,13,14]. In addition, the improved survival of patients originally with complex CHD lesions, who were previously associated with high mortality during childhood, may have further contributed to the increase in ACHD patients with severe conditions [15].

Despite the need for continuous specialist follow-up, many ACHD patients experience interruptions or discontinuity in care, especially during the transition from pediatric to adult medical services. Prior studies from Europe and North America have demonstrated that gaps in specialist ACHD care significantly worsen clinical outcomes, increasing rates of emergency hospital admissions and mortality, which may have been co-founded by socioeconomic circumstances in various healthcare systems within such countries [5,16,17,18,19,20,21,22]. However, comprehensive national data evaluating the relationship between emergency or non-referral admissions and acute outcomes, particularly within a nationwide universal healthcare system context, remain limited.

Japan offers a unique context to address these gaps due to its universal healthcare coverage and recently established structured ACHD specialist certification system [23,24]. Moreover, Japan’s rapidly aging society mirrors demographic trends seen in other developed countries, providing valuable insights into the evolving epidemiology and healthcare demands of ACHD patients [25]. Previous reports estimate over 500,000 ACHD patients in Japan, and this number is steadily increasing [26,27]. Recognizing these demographic shifts, Japan implemented formal transition care guidelines and specialist certification programs starting in 2018–2019 [24,27]. In addition, in Japan, we have the Japanese Registry of All Cardiac and Vascular Diseases–Diagnosis Procedure Combination (JROAD-DPC) database, which allowed us to analyze ACHD hospitalizations across hundreds of hospitals under Japan’s universal healthcare coverage and standardized Diagnosis Procedure Combination (DPC) coding system [23,25]. This unique dataset allows a systematic evaluation of ACHD admissions in a setting that combines nationwide universal healthcare access with a recent but formalized ACHD specialist system [28]. However, the impact of these initiatives on continuity of care, hospital admission types, and clinical outcomes has not been evaluated [28,29,30].

Given this background, we hypothesized that despite universal healthcare coverage and a formal specialist ACHD care framework, substantial numbers of ACHD patients still experience disrupted care, reflected by high rates of emergency and non-referral admissions. In addition, we expected that older age, increasing CHD complexity, and admission at non-specialist ACHD centers would further exacerbate adverse clinical outcomes. In the present study, using JROAD-DPC database, we aimed to characterize epidemiological trends, clinical outcomes, and predictors of in-hospital mortality utilization among ACHD patients hospitalized for cardiovascular comorbidities.

## 2. Methods

### 2.1. Study Design and Data Source

This was a retrospective, nationwide cohort study using the JROAD-DPC database, a comprehensive administrative claims dataset covering hospital admissions across Japan. The JROAD-DPC database includes standardized discharge data from hospitals participating in the DPC/Per Diem Payment System under Japan’s universal health coverage [25]. This registry captures detailed clinical and procedural information, diagnostic codes (ICD-10), hospital characteristics, patient demographics, admission pathways, and in-hospital outcomes. Japanese universal health coverage was established in 1961, achieving nearly complete insurance coverage to all citizens, standardized reimbursement fees, and equitable healthcare access regardless of socioeconomic status or background [23]. The study was conducted in accordance with the Declaration of Helsinki and approved by The Ethics Committee of the National Cardiovascular Center (Osaka, Japan) (protocol code R20021-5 and Approval Date: 7 July 2020).

### 2.2. Study Population

**Figure 1:**
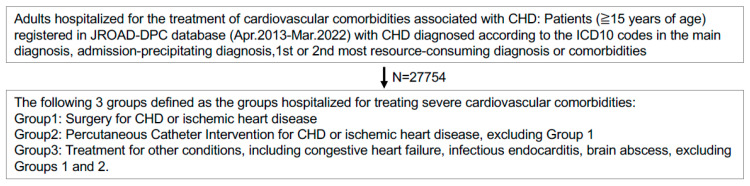
Flow Diagram. CHD: congenital heart disease; JROAD-DPC: Japanese Registry of All Cardiac and Vascular Diseases–Diagnosis Procedure Combination.

We identified adult patients (aged ≥ 15 years) hospitalized primarily for cardiovascular complications associated with congenital heart disease (CHD) between April 2013 and March 2022. Patients were included if CHD was recorded as the primary admission diagnosis, admission-precipitating diagnosis, the first or second resource-consuming condition or comorbidities during hospitalization (Figure 1). Disease severity was classified according to the 2001 American College of Cardiology/American Heart Association (ACC/AHA) guidelines and the Japanese Circulation Society (JCS) guideline into simple, moderate, or severe categories (Supplemental Method S1) [9,27,31]. Patients younger than 15 years or with incomplete admission data were excluded. Certified ACHD specialist hospitals were defined according to Japanese Society for Adult Congenital Heart Disease (JSACHD) certification criteria, which include 86 facilities accredited by adult and pediatric cardiology societies to provide specialized ACHD care, as of February 2023 [24]. The primary diagnosis, procedure records, and surgical records in the DPC database were previously validated [32,33,34].

### 2.3. Classification of Treatment Groups

Patients were categorized into three mutually exclusive groups based on specific interventions, procedures and ICD 10 codes, recorded during hospitalization (Figure 1) (Supplemental Method S2): Surgical intervention group: patients undergoing surgical procedures for CHD repair or coronary artery bypass grafting (CABG); Catheter intervention group: patients undergoing catheter-based interventions for CHD or ischemic heart disease; Medical treatment group: patients receiving medical management only (e.g., heart failure, arrhythmias, infective endocarditis, brain abscess, pulmonary hypertension, out of hospital cardiac arrest) without surgical or catheter intervention. Patients who underwent both surgical and catheter procedures were classified in the surgical intervention group.

### 2.4. The Outcome and Admission Types (Emergency and Non-Referral Admissions)

The primary outcome was in-hospital mortality. Emergency admissions were identified using standardized DPC codes (code 200 for unscheduled admission, code 3## for emergency admission, or ambulance transportation records). Non-referral admissions were defined as hospitalizations without formal referrals from any medical institutions, reflecting potential disruptions in specialist follow-up care.

### 2.5. Statistical Analysis

Continuous variables were summarized using medians with interquartile ranges (IQR), and categorical variables were reported as the numbers and percentages. Comparisons between groups were conducted using the rank-sum test or Kruskal–Wallis tests for continuous variables and chi-square tests for categorical variables. Univariate and multivariable multilevel mixed effects logistic regression models with the institution as a random intercept were performed to estimate odds ratios (ORs) and 95% confidence intervals for in-hospital mortality and for emergency or non-referral admission. All hospital costs and charges were converted into US dollars according to the current exchange rate (1 US dollar = 150.00 yen). Multilevel logistic regression analyses were conducted to identify independent predictors of the primary outcome, adjusting for hospital-level clustering. Covariates included in the multivariable models were selected based on clinical relevance and statistical significance in univariate analyses, including age, gender, ACHD center, CHD complexity, and admission pathway (emergency/non-referral). Multivariable analysis was conducted with two or three models for the in-hospital mortality (as in the models 1, 2 and 3), as well as for non-referral or emergency admission (as in the models 1 and 2) in the overall admission or in a subgroup. Multicollinearity was assessed and managed by separately modeling variables with high correlation, as necessary. All statistical analyses were conducted using SAS 9.4 (SAS Institute, Cary, NC, USA) and STATA 16.1 (College Station, TX, USA).

### 2.6. Ethics Approval

The study protocol conforms to the ethical guidelines of the 1975 Declaration of Helsinki. This study protocol was approved by the Ethics Committee of Mie University Hospital (approval number H2022-201) and the Institutional Review Board of the National Cerebral and Cardiovascular Center (approval number R20021-4). Given the retrospective nature and anonymized use of administrative data, individual informed consent was waived, consistent with Japanese ethical guidelines. Patients were informed about the study through institutional websites and posters, with an opt-out provision clearly provided.

## 3. Results

### 3.1. Patient Characteristics

We analyzed a total of 27,754 ACHD admissions (median age, 59 years; IQR, 36–74; 49% male) during the study period (Table 1). The median body mass index (BMI) was 21.6 (IQR, 19.2–24.4) and 49% of patients were male. Patients were categorized into surgical intervention (31.7%, *n* = 8800), catheter-based intervention (11.0%, *n* = 3060), and medical treatment groups (57.3%, *n* = 15,894). Emergency admissions accounted for 35.2%, while non-referral admissions comprised 9.9%. Regarding CHD complexity, admissions consisted of 60.7% simple, 23.2% moderate, and 9.4% severe cases (Supplemental Table S1). Certified ACHD specialist centers handled 43.8% of admissions.

**Figure 2:**
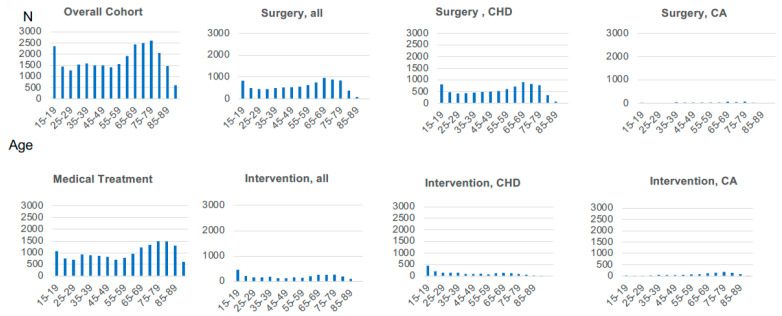
Age Distribution of overall admissions and treatment groups. CHD: congenital Heart Disease; CA: Coronary Artery; N: number of patients.

The age distribution showed a distinct bimodal pattern, featuring an initial peak among younger patients aged 15–25 years and a more prominent peak among older patients aged 65–84 years, reflecting demographic and generational shifts (Figure 2). Admissions for surgical and catheter-based interventions for CHD displayed clear bimodal peaks, whereas admissions for ischemic heart disease interventions and medical treatments gradually increased with age (Figure 2 and Supplemental Figure S1). Further demographic details comparing groups and admission status types are presented in Supplemental Tables S2 and S3 and Supplemental Results.

### 3.2. Predictors of Mortality and Emergency and Non-Referral Admissions

Multivariable logistic regression identified older age (OR, 1.02; 95% CI, 1.017–1.024; *p* < 0.001 in Model 1 and OR, 1.014; 95% CI, 1.011–1.017; *p* < 0.001 in Model 2), moderate-to-severe CHD complexity (OR, 1.775; 95% CI, 1.580–1.994; *p* < 0.001 in Model 1 and OR, 1.488; 95% CI, 1.324–1.671; *p* < 0.001 in Model 2), admission to non-specialist hospitals (OR, 0.54; 95% CI, 0.439–0.665; *p* < 0.001 in Model 1 and OR, 0.752; 95% CI, 0.633–0.893; *p* = 0.001 in Model 2), emergency admissions (OR, 8.67; 95% CI, 7.406–10.057; *p* < 0.001 in Model 2), and non-referral admissions (OR, 2.354; 95% CI, 2.040–2.717; *p* < 0.001 in Model 1) as significant independent predictors of increased mortality (Table 2 and Table 3). Furthermore, older age, greater CHD complexity, and admission to non-specialist hospitals were significant predictors of both emergency and non-referral admissions (all *p* < 0.001; Supplemental Table S4). These associations remained consistent within the subgroup of medically managed patients.

## 4. Discussion

In the present study, we demonstrated that emergency and non-referral admissions independently predict increased mortality among ACHD patients hospitalized with cardiovascular comorbidities. Despite universal healthcare coverage and a formalized ACHD specialist network in Japan, these disruptions in specialist care remain prevalent and substantially impact outcomes. We further identified a notable bimodal age distribution of patients, reflecting the generational effects of recent advancements in pediatric cardiac care on an aging patient population requiring increasingly complex clinical management.

### 4.1. Emergency and Non-Referral Admission as the Risk for In-Hospital Mortality

Our findings highlighted that a significant proportion of ACHD hospitalizations occurred via emergency (35%) or non-referral (9.9%) pathways, both strongly associated with higher in-hospital mortality. Similar patterns have been observed in other developed countries, where disrupted specialist care similarly correlated with increased morbidity and mortality [7,19,35]. Such disrupted special care may have been influenced by diverse insurance coverage and variable specialist availability [5,6,16,20,36]. Our results uniquely extend these observations by demonstrating in a nationwide manner that even comprehensive universal healthcare alone cannot fully mitigate gaps in ACHD specialist care without structured, system-wide interventions. These findings underscore that disrupted specialist care or loss to follow-up is not merely administrative but rather a critical, modifiable clinical risk factor. Strengthening structured transition programs with appropriate referral networks and addressing non-financial barriers, such as geographic and provider availability limitations, may be required for reducing emergency and non-referral admissions [5,22,37,38].

### 4.2. Aging ACHD Population and Generational Shift

The aging trend observed in the ACHD population in Japan, with a median age approaching 60 years and a bimodal distribution (15–25 and 65–84 years), reflects broader demographic shifts, increased survival due to advancements in pediatric cardiology, and evolving clinical management systems [11,39,40,41]. Of interest, subgroup analysis revealed a gradual increase in admissions for surgical and interventional procedures for CHD with patient aging, as well as an age-dependent increase in admissions for heart failure and ischemic heart disease treatments. Similar age distributions have been reported in the United States and Europe, where improvements in congenital heart surgery and medical management have led to an expanding cohort of elderly ACHD patients requiring complex, long-term care [11,14,27,28,40,41]. Despite improved survival, older ACHD patients exhibit significant morbidity and are at increased risk for cardiovascular complications, particularly heart failure, arrhythmias, and ischemic heart disease [2,5,12,39,40,41]. Age-related factors, including systemic hypertension, diabetes, and chronic kidney disease, may further exacerbate their vulnerability [11,12,19]. In Japan, where life expectancy is among the highest globally, this aging trend necessitates heightened surveillance and proactive management of multimorbidity in ACHD patients [28,29]. Heart failure, in particular, has emerged as a dominant cause of morbidity and mortality in this population. Several large-scale studies have reported that heart failure-related admissions in ACHD patients have increased significantly over the past two decades, often necessitating intensive care [28,42,43].

In addition, we observed a younger peak (15–25 years of age) in admissions for surgical and interventional procedures for CHD, in contrast with earlier studies, which primarily focused on older adults, leaving generational differences and the impact of recent pediatric advances largely unexplored. This initial peak in the present study may reflect the ongoing need for management of moderate or severe CHD during late adolescence and early adulthood, consistent with previous findings [29,44]. Furthermore, this younger peak can be explained by generational factors and evolving population-level effects, including systematic improvements in pediatric diagnostic and surgical interventions over recent decades in Japan [26,45]. Consequently, patients currently around 20 years of age represent the severe CHD generation systematically diagnosed and managed from childhood, suggesting the age of peak severity may gradually shift upward as this cohort ages. Moderate and severe CHD lesions, such as conotruncal defects or single-ventricle physiology, are expected to increasingly contribute to the burden of surgical or interventional procedures [45]. Therefore, early risk stratification and lifelong management strategies remain critical to address the anticipated evolving clinical needs of these patients [7,21,46,47]. Thus, healthcare systems, particularly in rapidly aging societies like Japan and the United States, must anticipate this shift by expanding specialized ACHD resources and enhancing collaboration between ACHD specialists, general cardiologists, and geriatric care providers [35].

In this nationwide claims-based study, we first characterized ACHD patients hospitalized for comprehensive cardiovascular comorbidities in Japan and identified several factors associated with worse in-hospital outcomes. A significant strength of this study is the use of the comprehensive JROAD-DPC database, enabling systematic evaluation across numerous hospitals under Japan’s universal healthcare system and standardized coding framework [23,25]. This unique dataset provides generalizable insights into continuity of care and acute outcomes within a structured ACHD specialist care network.

## 5. Limitations

Several limitations should be acknowledged. First, as this was a retrospective study using administrative data, which do not include ultrasound data, misclassification and residual confounding may exist despite the high accuracy of diagnoses and procedures in validation studies. Second, the analysis focused solely on in-hospital outcomes, without data on longer-term outcomes post-discharge. Third, our definitions of disrupted specialist care (emergency and non-referral admissions) were proxies and may not fully represent actual patient follow-up behaviors: these parameters are indirect indicators and may be influenced by unmeasured clinical severity or social factors. Fourth, specialist center designations were determined based on recent certifications, possibly not capturing variations in specialist availability throughout the study period. Limitations in such designations may influence the analysis of the role of specialist centers. Fifth, teaching hospitals form the majority of JROAD-DPC participants, potentially skewing the sample toward more severe cases. Sixth, in the JROAD-DPC dataset, which limits the perfect linking of patients across different fiscal years or hospital transfers, the database included repeated admissions of the same patient as separate events. Although repeated admissions from the same patient may occur, the analyses were conducted at the admission level with institution-level random effects, which is appropriate for population-level inference in administrative multicenter databases. It is still possible that such limitations may influence the severity of overall cases and outcome prediction analysis. Seventh, preconception factors and the use of new pharmacotherapy for heart failure which could have an impact on the outcomes of CHD patients were not investigated because of the lack of such data [1,48]. 

## 6. Implications

First, our findings showed that indicators of disrupted specialist follow-up, specifically, emergency and non-referral admissions, are associated with adverse outcomes, even within a universal healthcare system. Strengthening structured transition-of-care programs and improving integration between pediatric and adult cardiology services could significantly mitigate these acute events [6,36]. Second, given the increasing population of older and moderate-to-severe ACHD patients, collaboration among general cardiologists, ACHD specialists, and geriatric/internal medicine providers is essential to comprehensively address their evolving healthcare needs. Finally, ongoing research into effective risk stratification may help optimize resource allocation, particularly for older patients or those with complex congenital heart defects, who demonstrate notably higher rates of hospital readmission and mortality [16,35].

## 7. Conclusions

Our findings showed emergency and non-referral admissions as critical and potentially modifiable predictors of adverse outcomes in ACHD patients, even within nationwide universal healthcare systems. Enhanced structured transition programs, improved specialist care accessibility, and integrated ACHD care networks, along with heightened clinical awareness of ACHD in the elderly, may contribute to optimizing clinical outcomes for this increasingly complex and aging patient population internationally, as well as in Japan, where the networking of specialist centers is still developing.

## Figures and Tables

**Table 1:** Demography.

Groups	Overall	Surgery	Surgery	Surgery	Intervention	Intervention	Intervention	Medical Treatment
		All	:CHD	:Coronary	All	:CHD	:Coronary	
N	27,754	8800	8343	457	3060	1998	1062	15,894
Age (median, year)	59	56	55	60	53	37	70	62
(IQR)	36.0, 74.0	34.0, 69.0	34.9, 69.0	42.0, 72.0	27.0, 71.0	21.0, 62.0	56.0, 78.0	38.0, 78.0
Male (%)	13,635 (49.1)	4443 (50.5)	4117 (49.3)	7326 (1.3)	1503 (49.1)	785 (39.3)	718 (67.6)	7689 (48.4)
Body mass index (median)	21.6	21.9	21.8	23.5	21.8	21	23.3	21.5
(IQR)	19.2, 24.4	19.6, 24.4	19.5, 24.3	21.4, 26.0	19.4, 24.6	18.8, 23.8	21.0, 25.7	18.9, 24.3
Smoking (%)	6118 (22.0)	2245 (25.5)	2975 (24.9)	170 (37.2)	630 (20.6)	213 (10.7)	417 (39.3)	4243 (20.4)
Treatment								
HF or ARR (%)	25,569 (92.1)	8772 (99.7)	8316 (99.7)	456 (99.8)	1275 (41.7)	555 (27.8)	720 (67.8)	15,522 (97.7)
Heart Failure (%)	24,988 (90.0)	8736 (99.3)	8282 (99.3)	454 (99.3)	835 (27.9)	293 (14.7)	560 (52.7)	15,399 (96.9)
Arrhythmia (%)	17,342 (62.5)	7922 (90.0)	7478 (89.6)	444 (97.2)	829 (27.1)	360 (18.0)	469 (44.2)	8591 (54.1)
PH therapy(%)	2428 (8.7)	324 (3.7)	321 (3.8)	3 (0.7)	528 (17.3)	514 (25.7)	14 (1.3)	1576 (9.9)
OHCA(%)	236 (0.9)	4 (<0.1)	2 (<1.0)	2 (0.4)	12 (0.4)	0 (0)	12 (1.1)	220 (1.4)
Infectious endocarditis(%)	802 (2.9)	234 (2.7)	234 (2.8)	0 (0)	1 (<0.1)	1 (0.1)	0 (0)	567 (3.6)
Brain abscess(%)	20 (0.1)	1 (<0.001)	1 (<1.0)	0 (0)	0 (0)	0(0)	0 (0)	19 (0.1)
Oral medication at discharge								
Anti-hypertension	17,290 (62.3)	7032 (79.9)	6672 (80.0)	360 (78.8)	1242 (40.6)	696 (34.8)	546 (51.4)	9016 (56.7)
Anti-diabetes	1651 (5.9)	622 (7.1)	562 (6.7)	60 (13.1)	134 (4.4)	29 (1.5)	105 (9.9)	895 (5.6)
Anticoagulant	11,916 (43.0)	5616 (63.8)	5373 (65.1)	187 (40.9)	703 (23.0)	510 (25.6)	193 (18.2)	5598 (35.2)
Warfarin	8716 (31.4)	5120 (58.2)	4970 (59.6)	150 (32.8)	387 (12.6)	302 (15.1)	85 (8.0)	3209 (20.2)
DOAC/NOAC	3203 (11.5)	496 (5.6)	459 (5.5)	37 (8.1)	317 (10.4)	209 (10.5)	108 (10.2)	2390 (15.0)
Anti-platelet	13,079 (47.1)	5083 (57.8)	4692 (56.2)	391 (85.6)	1732 (56.6)	859 (43.0)	873 (82.2)	6264 (39.4)
Aspirin	7092 (25.6)	3402 (38.7)	3040 (36.4)	362 (79.2)	1099 (35.9)	418 (20.9)	681 (64.1)	2591 (16.3)
Other anti-platelet	8795 (31.7)	2763 (31.4)	2603 (31.2)	160 (35.0)	1441 (47.1)	621 (31.1)	820 (77.2)	4591 (28.9)
DAPT	2808 (10.1)	1082 (12.3)	951 (11.4)	131 (28.7)	808 (26.4)	180 (9.0)	628 (59.1)	918 (5.8)
Statin	3303 (11.9)	1196 (13.6)	977 (11.7)	219 (47.9)	642 (21.0)	133 (6.7)	509 (47.9)	1465 (9.2)
Socioeconomic parameters								
Hospital stay (median days)	21	21	21	5	5	5	13	
(IQR days)	7.0, 25.0	16.0, 29.0	16.0, 29.0	16.0, 30.0	4.0, 9.0	4.0, 7.0	3.0, 13.0	6.0, 24.0
Hospital charge (USD)	12,041.30	28,360.00	28,536.70	25,250.70	6735.30	6082.70	8636.70	6434.00
Hospital parameters								
CVIT Center (%)	24,620 (88.7)	8324 (94.6)	7891 (94.6)	433 (94.7)	2768 (90.5)	1854 (92.8)	914 (86.1)	13,528 (85.1)
ACHD Center (%)	12,168 (43.8)	4399 (50.0)	4231 (50.7)	168 (36.8)	1592 (52.0)	1352 (67.7)	240 (22.6)	6177 (38.9)
Beds (N)	612	642	644	608	612	663	455	581
Mode of hospitalization								
Emergency (%)	9759 (35.2)	779 (8.9)	693 (8.3)	86 (18.8)	438 (14.3)	47 (2.3)	391 (39.4)	8542 (53.7)
No Referral (%)	2750 (9.9)	495 (5.6)	439 (5.3)	56 (12.3)	274 (9.0)	81 (4.1)	193 (18.2)	1981 (12.5)
Outcome parameters								
ICU care (%)	11,213 (40.4)	7920 (90.0)	7510 (90.0)	410 (89.7)	364 (11.9)	214 (10.7)	150 (14.2)	2929 (18.4)
Hospital mortality (%)	1392 (5.0)	144 (1.6)	129 (1.5)	15 (3.3)	34 (1.1)	6 (0.3)	28 (2.6)	1214 (7.6)

IQR: Interquartile range; HF: heart failure; ARR: arrhythmia; PH: pulmonary hypertension; OHCA: out-of-hospital cardiac arrest; DOAC: Direct Oral Anticoagulant; NOAC: novel oral anticoagulant; DAPT: Dual Anti-Platelet Therapy; CVIT: Japanese Association of Cardiovascular Intervention and Therapeutics; ACHD: adult congenital heart disease; ICU: intensive care unit.

**Table 2:** Univariate and multivariable analyses for hospital death in overall admissions and in the medical treatment group.

Overall admissions									
**Variables**	**Univariate**			**Multivariable model 1**			**Multivariable model 2**		
	OR	95% CI	*p*	OR	95% CI	*p*	OR	95% CI	*p*
Age	1.024	1.021–1.027	<0.001	1.02	1.017–1.024	<0.001	1.014	1.011–1.017	<0.001
Male Gender	0.95	0.849–1.063	0.371	1.086	0.969–1.217	0.2156	1.108	0.987–1.243	0.083
ACHD center	0.35	0.28–0.438	<0.001	0.54	0.439–0.665	<0.001	0.752	0.633–0.893	0.001
Emergency	10.31	8.863–11.993	<0.001				8.64	7.406–10.057	<0.001
B or C	1.884	1.676–2.118	<0.001	1.775	1.580–1.994	<0.001	1.488	1.324–1.671	<0.001
No referral	2.755	2.385–3.183	<0.001	2.354	2.040–2.717	<0.001			
**The medical treatment group**									
**Variables**	**Univariate**			**Multivariable model 1**			**Multivariable model 2**		
	OR	95% CI	*p*	OR	95% CI	*p*	OR	95% CI	*p*
Age	1.023	1.020–1.026	<0.001	1.02	1.017–1.023	<0.001	1.014	1.011–1.017	<0.001
Male Gender	0.95	0.850–1.063	0.374	1.09	0.973–1.222	0.138	1.115	0.993–1.252	0.065
ACHD center	0.374	0.300–0.466	<0.001	0.564	0.461–0.691	<0.001	0.755	0.636–0.897	0.001
Emergency	9.248	7.950–10.755	<0.001				7.685	6.595–8.955	<0.001
B or C	1.983	1.764–2.229	<0.001	1.869	1.664–2.100	<0.001	1.526	1.358–1.715	<0.001
No referral	2.689	2.329–3.106	<0.001	2.281	1.977–2.632	<0.001			

ACHD: adult congenital heart disease; B: moderate grade of congenital heart disease; C: severe grade of congenital heart disease.

**Table 3:** Univariate and multivariable analyses for non-referral or emergency admission in overall admissions and the medical treatment group.

Overall admissions						
**No referral admission**						
**Variables**	**Univariate**			**Multivariable**		
	OR	95% CI	*p*	OR	95% CI	*p*
Age	1.007	1.005–1.009	<0.001	1.006	1.004–1.008	<0.001
Male gender	0.981	0.899–1.071	0.671	1.022	0.936–1.117	0.628
ACHD center	0.252	0.181–0.351	<0.001	0.28	0.201–0.389	<0.001
B or C	1.408	1.281–1.547	<0.001	1.411	1.284–1.550	<0.001
**Emergency admission**						
**Variables**	**Univariate**			**Multivariable**		
	OR	95% CI	*p*	OR	95% CI	*p*
Age	1.015	1.013–1.016	<0.001	1.015	1.013–1.017	<0.001
Male gender	0.952	0.901–1.007	0.087	1.018	0.962–1.078	0.53
ACHD center	0.216	0.168–0.277	<0.001	0.279	0.220–0.353	<0.001
B or C	1.791	1.684–1.905	<0.001	1.87	1.757–1.990	<0.001
**The medical treatment group**						
**No referral admission**						
**Variables**	**Univariate**			**Multivariable**		
	OR	95% CI	*p*	OR	95% CI	*p*
Age	1.007	1.005–1.010	<0.001	1.006	1.004–1.009	<0.001
Male gender	0.954	0.872–1.04	0.03	0.999	0.912–1.094	0.98
ACHD center	0.261	0.188–0.362	<0.001	0.29	0.209–0.402	<0.001
B or C	1.469	0.998–0.999	<0.001	1.87	1.757–1.990	<0.001
**Emergency admission**						
**Variables**	**Univariate**			**Multivariable**		
	OR	95% CI	*p*	OR	95% CI	*p*
Age	1.015	1.013–1.016	<0.001	1.015	1.013–1.016	<0.001
Male gender	0.935	0.883–0.990	0.021	0.999	0.943–1.060	0.998
ACHD center	0.225	0.175–0.289	<0.001	0.288	0.227–0.365	<0.001
B or C	1.963	1.884–2.092	<0.001	2.051	1.924–2.188	<0.001

ACHD: adult congenital heart disease; B: moderate grade of congenital heart disease; C: severe grade of congenital heart disease.

## Data Availability

The data underlying this article cannot be shared publicly for the privacy of individuals that participated in the study. The data will be shared on reasonable request to the corresponding author.

## Supplementary Materials

The following supporting information can be downloaded at: https://www.mdpi.com/article/10.3390/healthcare14030315/s1, Table S1. Underlying Cardiac Diseases by the Complexity (*n* = 27,754). Table S2. Comparison between the Surgery+ Catheter Intervention groups and the medical treatment groups. Table S3. Emergency admission and non-referral admission in overall groups and the medical treatment group. Table S4. Univariate and multivariable analyses for no-referral or emergency admission in overall admissions and the medical treatment group. Figure S1. Age Distribution (%). Supplemental Method S1. Complexity A (Simple), Complexity B (Moderate) and Complexity C (Severe). Supplemental Method S2. Procedure code. Supplemental Results.

## Abbreviations

ACHD	adult congenital heart disease
CHD	congenital heart disease
JROAD-DPC	Japanese Registry of All Cardiac and Vascular Diseases–Diagnosis Procedure Combination
BMI	body mass index
DPC	Diagnosis Procedure Combination
ICD	International Statistical Classification of Diseases and Related Health Problems
ACC	American College of Cardiology
AHA	American Heart Association
JCS	Japanese Circulation Society
JSACHD	Japanese Society for Adult Congenital Heart Disease
CABG	coronary artery bypass grafting
