# Supplementary material for: Emergency and Non-Referral Admissions as Predictors of Hospital Mortality Among Adults with Congenital Heart Diseases: A Nationwide Claim-Based Registry Study in Japan
Source: Healthcare (Basel). 2026 Jan 27;14(3):315. doi: 10.3390/healthcare14030315 (PMC12896941; doi:10.3390/healthcare14030315)
Supplement: Supplementary file 1 [file healthcare-14-00315-s001.zip › healthcare-4087224-supplementary/suppl files/Supplemental Method S1.pdf]

Supplemental Method S1

**Complexity A (Simple):**	**ICD Code**
**A-1.** Atrial septal defect	Q211
**A-2.** Ventricular septal defect	Q210
**A-3.** Patent ductus arteriosus	Q250
**A-4.** Aortic diseases including left ventricular outflow tract obstruction (bicuspid aortic valve, congenital supravalvular, valvular, or subvalvular aortic stenosis)	Q253
**Complexity B (Moderate):**	
**B-1.** Atrioventricular septal defect	Q212
**B-2.** Coarctation or interruption of the aorta	Q251
**B-3.** Right ventricular outflow tract obstruction diseases (two-chambered right ventricle, congenital supravalvular, valvular, or subvalvular pulmonary stenosis)	Q243.Q256
**B-4.** Cor triatriatum	Q242
**B-5.** Partial anomalous pulmonary venous return	Q263, Q264
**B-6.** Total anomalous pulmonary venous return	Q262
**B-7.** Ebstein's anomaly	Q225
**B-8.** Absent pulmonary valve syndrome	Q223
**B-9.** Tetralogy of Fallot	Q213. Q215
**B-10.** Double outlet right ventricle	Q201
**B-11.** Coronary artery anomalies (coronary arteriovenous fistula, Bland-White-Garland syndrome, anomalous origin of coronary arteries)	Q245
**Complexity C (Severe):**	
**C-1.** Truncus arteriosus	Q200
**C-2.** Pulmonary atresia with intact ventricular septum	Q220, Q255, Q226.
**C-3.** Congenitally corrected transposition of the great arteries	Q205
**C-4.** Complete transposition of the great arteries	Q203
**C-5.** Single ventricle	Q204
**C-6.** Tricuspid atresia	Q224
**C-8.** Hypoplastic left heart syndrome	Q234, Q252
**C-9.** Fontan circulation	I 971
**C-10.** Congenital Heart Disease associated with pulmonary hypertension	I 278
