# Supplementary material for: Emergency and Non-Referral Admissions as Predictors of Hospital Mortality Among Adults with Congenital Heart Diseases: A Nationwide Claim-Based Registry Study in Japan
Source: Healthcare (Basel). 2026 Jan 27;14(3):315. doi: 10.3390/healthcare14030315 (PMC12896941; doi:10.3390/healthcare14030315)
Supplement: Supplementary file 1 [file healthcare-14-00315-s001.zip › healthcare-4087224-supplementary/suppl files/Supplemental Method S2.pdf]

Supplemental Method S2

1 Procedure codes for the surgery and catheter intervention were defined as follows:

1) Surgery

For CHD:

valvuloplasty (K5541-3), valve replacement (K5551-3), surgery for AS (K556, K557-2,K557-3, K558, K567-1), PDA (K5631-22), CoA (K5671-3), PS (K5701-2), ASD (K5741-2), VSD (K5761-4), TOF (K5801-2), CoA (K5671-3), PA banding (K563), systemic pulmonary shunt (K566), AVSD (K5791-2), PAPVR (K5711), TCRV (K578), DORV (K5821-2), cTGA (K5841-2), Ebstein anomaly (K569), Valsalva aneurysm (K5771-2), Anomalous origin of CA (K589), Ross operation (K558), Glen procedure (K5861), Fontan Procedure (K5862)

For Ischemic heart disease:

balloon angioplasty (K5461-3), stenting (K5491-3), rotavolator (K547), directional coronary atherectomy (K5481), Exima laser (K5482), intracoronary thrombolysis (K550), or aspiration thrombectomy (K550-2)

2) Catheter intervention

For congenital heart disease (CHD):

PDA closure (K5621), AS (K567-2), ASD(K574-2). Valvular PS (K570-2), PS (K570-3), angioplasty (150347310)

For ischemic heart disease (CABG):

codes K5521, K5522, K552-21, or K552-22

2 In-hospital treatments for the medical treatment group, including for heart failure, arrhythmia, pulmonary hypertension, infectious endocarditis, brain abscess and out-of-hospital cardiac arrest. and ICU care as define as follows:

1) **In-hospital treatment for heart failure:** defined by the presence of any of the following treatments: intravenous administration of catecholamines, diuretics (furosemide), vasodilators (human natriuretic peptide, milrinone, nitroglycerin), intra-aortic balloon pump, percutaneous cardiopulmonary support, ventricular assist device, or percutaneous ventricular assist device (Impella).

2) **In-hospital treatment for arrhythmia**: defined by the presence of the in-hospital administration of anti-arrhythmic compounds (class 1, II, III, IV), or aβ blocker (landiolol), treatments with the defibrillation, ablation, implantable cardioverter defibrillator or pacemaker.

3) **In-hospital treatment for pulmonary hypertension**: defined by the presence of the in-hospital administration of endothelin receptor antagonists, phosphodiesterase inhibitors, guanylate cyclase activators, prostanoids.

4) **In-hospital treatment for infectious endocarditis:** defined by the presence of the ICD 10 code I330, recorded as the primary admission diagnosis, admission-precipitating diagnosis, the first or second resource-consuming condition.

5) **In-hospital treatment for brain abscess** : defined by the presence of the ICD 10 code G060, recorded as the primary admission diagnosis, admission-precipitating diagnosis, the first or second resource-consuming condition.

6) **In-hospital treatment for out-of-hospital cardiac arrest**: defined by the presence of the ICD 10 code I46.0 and I46.1, recorded as the primary admission diagnosis, admission-precipitating diagnosis, the first or second resource-consuming condition.

Other procedure codes for covariants

1) **Intensive care unit (ICU)care** : defined by specific DPC billing codes for intensive or high-care units (codes A301-00 or A301-02).
