# Supplementary material for: Emergency and Non-Referral Admissions as Predictors of Hospital Mortality Among Adults with Congenital Heart Diseases: A Nationwide Claim-Based Registry Study in Japan
Source: Healthcare (Basel). 2026 Jan 27;14(3):315. doi: 10.3390/healthcare14030315 (PMC12896941; doi:10.3390/healthcare14030315)
Supplement: Supplementary file 1 [file healthcare-14-00315-s001.zip › healthcare-4087224-supplementary/suppl files/Supplemental Results.pdf]

Supplemental Results

Compared with the combined Surgery and catheter intervention groups, a subgroup, the medical treatment group, was characterized by the older age, admission to non-teaching centers, the lower hospital bed counts, higher percentage of emergency and non-referral admissions, and the in-hospital death (all <.001) (Table S2). Compared with referral admissions and non-emergency admissions, non-referral admission or emergency admissions were characterized by the older age, admission to non-teaching centers, the lower hospital bed counts, higher percentage of emergency and non-referral admissions, and the in-hospital death (all <.001) (Table S3).
