# Supplementary material for: Emergency and Non-Referral Admissions as Predictors of Hospital Mortality Among Adults with Congenital Heart Diseases: A Nationwide Claim-Based Registry Study in Japan
Source: Healthcare (Basel). 2026 Jan 27;14(3):315. doi: 10.3390/healthcare14030315 (PMC12896941; doi:10.3390/healthcare14030315)
Supplement: Supplementary file 1 [file healthcare-14-00315-s001.zip › healthcare-4087224-supplementary/suppl files/Table S1.pdf]

Table S1 **Underlying Cardiac Diseases by the Complexity** (n=27,754)

Simple

ASD 10,948(39.45%),	VSD, 3,462(12.47%)	PDA, 1,221(4.40%)	AS, 1,826(4.40%)

Moderate

AVSD, 816(2.94%)	CoA/IAA, 1,826(6.58%)	RVOTS, PS, 1,557(5.61%)	Aortic disease, 51(.18%)
Cor Triatriatum, 69(.25%)	PAPVR, 82(.30%)	TAPVR, 70(.25%)	Ebstein anomaly, 384(1.40%)
TOF, 245(.88%)	DORV, 773(2.79%)	Coronary Anomaly, 570(2.05%)	

Severe

TAC, 15(.05%)	PA/IVS 506(1.82%)	ccTGA, 693(2.50%)	SV, 1,308(4.71%)
PH-CHD 99(.36%)			

Complexity A: simple CHD; Complexity B: moderate; complexity C: severe

ASD: atrial septal defect; VSD: ventricular septal defect; PDA: patent ductus arteriosus; AS: aortic stenosis; AVSD: atrioventirular septal defect; CoA: coarctation of aort; IAA: interruption of aortic arch; RCOTS: right ventricular outflow tract stenosis; PS: pulmonary stenosis; PAPVR: Partial anomalous pulmonary venous return; TAPVR: Total anomalous pulmonary venous return; TOF: Tetralogy of Fallot; DORV: Double outlet right ventricle; TAC: Truncus arteriosus; PA/IVS: Pulmonary atresia with intact ventricular septum; ccTGA: Congenitally corrected transposition of the great arteries; SV: Single ventricle; PH-CHD: Congenital Heart Disease associated with pulmonary hypertension
