# Supplementary material for: Emergency and Non-Referral Admissions as Predictors of Hospital Mortality Among Adults with Congenital Heart Diseases: A Nationwide Claim-Based Registry Study in Japan
Source: Healthcare (Basel). 2026 Jan 27;14(3):315. doi: 10.3390/healthcare14030315 (PMC12896941; doi:10.3390/healthcare14030315)
Supplement: Supplementary file 1 [file healthcare-14-00315-s001.zip › healthcare-4087224-supplementary/suppl files/Table S2.pdf]

Table S2 Comparison between the Surgery+ Catheter Intervention groups and the medical treatment groups

Groups	Surgery＋Catheter Intervention groups	Medical Treatment group	P value
N	11,860	15,894	
Age (median year)	55.0	62.0	<.001
(IQR)	(33.0, 70.0)	(38.0, 78.0)	
Male (%)	5946 (50.1)	7689 (48.4)	.004
BMI	21.9	21.5	.004
CVIT center (%)	11092 (93.5)	13528 (85.1)	<.001
ACHD center (%)	5991 (50.5)	6177 (38.9)	<.001
Beds (N)	635.0	581.0	<.001
Cardiology beds (N)	45.0	43.0	<.001
Emergency (%)	1217 (10.3)	8542 (53.7)	<.001
No referral (%)	769 (6.5)	1981 (12.5)	<.001
Hospital stay (median days)	18.0	13.0	<.001
Hospital Cost (median USD)	23608.9	6434.2	<.001
ICU management (%)	69.8 (69.8)	2929 (18.4)	<.001
Total hospital death (%)	178 (1.5)	1214 (7.6)	<.001
