# Supplementary material for: Emergency and Non-Referral Admissions as Predictors of Hospital Mortality Among Adults with Congenital Heart Diseases: A Nationwide Claim-Based Registry Study in Japan
Source: Healthcare (Basel). 2026 Jan 27;14(3):315. doi: 10.3390/healthcare14030315 (PMC12896941; doi:10.3390/healthcare14030315)
Supplement: Supplementary file 1 [file healthcare-14-00315-s001.zip › healthcare-4087224-supplementary/suppl files/Table S3.pdf]

Table S3 Emergency admission and non-referral admission in overall groups and the medical treatment group

Overall admission

	Total	Emergency	Non-emergency	P	Non-referral	Referral	P
(N)	27754	9754	17995		2750	25004	
Age (median year)	59.0	68.0	54.0	<.001	66.0	58.0	<.001
Male (%)	13635	4683 (48.0)	8952 (49.7)	.005	1334 (48.5)	12301 (49.2)	.49
CVIT center (%)	88.7	4683 (82.0)	16620 (94.5)	<.001	2248 (81.7)	22372 (89.5)	<.001
ACHD center (%)	43.8	2801 (28.7)	9367 (52.1)	<.001	809 (29.4)	11359 (45.4)	<.001
Hospital beds	612.0	512.0	644.0	<.001	481.0	612.0	<.001
Emergency (%)	35.2				1930 (70.2)	7829 (31.3)	<.001
Non-referral (%)	9.9	1930 (19.8)	820 (4.6)	<.001			
Hospital stay (median days)	16.0	18.0	14.0	<.001	17.0	16.0	<.001
Hospital cost (median USD)	12041.2	6926.0	16395.8	<.001	9073.8	12490.4	<.001
ICU care (%)	40.4	2067 (21.2)	9146 (50.8)	<.001	867 (31.5)	10346 (41.4)	<.001
Hospital mortality (%)	5.0	11173 (12.0)	219 (1.2)	<.001	326 (11.9)	1066 (4.3)	<.001

The medical treatment group

	Total	Emergency	Non-emergency	P	Non-referral	Referral	P
(N)	15894	8542	7352		1981	13913	
Age (median year)	62.0	69.0	55.0	<.001	69.0	61.0	<.001
Male (%)	48.4	3967 (46.4)	3722 (50.6)	<.001	887 (44.8)	6802 (48.9)	<.001
CVIT center (%)	85.1	6886 (80.6)	6642 (90.3)	<.001	1564 (79.0)	11964 (41.1)	<.001
ACHD center (%)	38.9	2443 (28.6)	3734 (50.8)	<.001	463 (23.4)	5714 (41.1)	<.001
Hospital beds	581.0	511.0	654.0	<.001	477.0	600.0	<.001
Emergency (%)	53.8				1629 (82.2)	6913 (49.7)	<.001
Non-referral (%)	12.5	1629 (19.1)	352 (4.8)	<.001			
Hospital stay (median days)	13.0	17.0	8.0	<.001	16.0	13.0	<.001
Hospital cost (median USD)	6434.2	6015.7	8260.4	.009	6639.7	6406.8	.091
ICU care (%)	18.4	1254 (14.7)	1675 (22.8)	<.001	383 (19.3)	2546 (18.3)	.27
Hospital mortality (%)	7.6	1099 (12.9)	115 (1.6)	<.001	300 (15.1)	914 (6.6)	<.001
