# Supplementary material for: Emergency and Non-Referral Admissions as Predictors of Hospital Mortality Among Adults with Congenital Heart Diseases: A Nationwide Claim-Based Registry Study in Japan
Source: Healthcare (Basel). 2026 Jan 27;14(3):315. doi: 10.3390/healthcare14030315 (PMC12896941; doi:10.3390/healthcare14030315)
Supplement: Supplementary file 1 [file healthcare-14-00315-s001.zip › healthcare-4087224-supplementary/suppl files/Table S4.pdf]

Table S4 **Univariate and multivariable analyses for no-referral or emergency admission in overall admissions and the medical treatment group**

Overall admissions

No referral admission

Variables	Univariate			Multivariable		
	OR	95%CI	P	OR	95%CI	P
Age	1.007	1.005-1.009	<.001	1.006	1.004-1.008	<.001
Male gender	.981	.899-1.071	.671	1.022	.936-1.117	.628
ACHD center	.252	.181-.351 .	<.001	.280	.201-.389 .	<.001
B or C	1.408	1.281-1.547	<.001	1.411	1.284-1.550	<.001

Emergency admission

Variables	Univariate			Multivariable		
	OR	95%CI	P	OR	95%CI	P
Age	1.015	1.013-1.016	<.001	1.015	1.013-1.017	<.001
Male gender	.952	.901-1.007	.087	1.018	.962-1.078	.53
ACHD center	.216	.168-.277	<.001	.279	.220-.353 .	<.001
B or C	1,791	1.684-1.905	<.001	1.87	1.757-1.990	<.001

The medical treatment group

No referral admission

Variables	Univariate			Multivariable		
	OR	95%CI	P	OR	95%CI	P
Age	1.007	1.005-1.010	<.001	1.006	1.004-1.009	<.001
Male gender	.954	.872-1.04	.030	.999	.912-1.094	.98
ACHD center	.261	.188-.362	<.001	.29	.209-.402	<.001
B or C	1.469	.998-.999	<.001	1.87	1.757-1.990	<.001

Emergency admission

Variables	Univariate			Multivariable		
	OR	95%CI	P	OR	95%CI	P
Age	1.015	1.013-1.016	<.001	1.015	1.013-1.016	<.001
Male gender	.935	.883-.990	.021	.999	.943-1.060	.998
ACHD center	.225	.175-.289	<.001	.288	.227-.365	<.001
B or c	1.963	1.884-2.092	<.001	2.051	1.924-2.188	<.001

ACHD: adult congenital heart disease; B: moderate grade of congenital heart disease; C: severe grade of congenital heart disease
